# Amino acid profile and oxidizable vitamin content of *Synsepalum dulcificum* berry (miracle fruit) pulp

**DOI:** 10.1002/fsn3.213

**Published:** 2015-03-12

**Authors:** Njideka E Njoku, Collins N Ubbaonu, Serah O Alagbaoso, Chioma N Eluchie, Munachiso C Umelo

**Affiliations:** Department of Food Science and Technology, Federal University of TechnologyOwerri, Nigeria

**Keywords:** Amino acid, berry, oxidizable vitamins, pulp, *Synsepalum dulcificum*

## Abstract

The amino acid profile of the *Synsepalum dulcificum* berry was studied. Among the essential amino acid observed, leucine (2.35 g/100 g protein) was the highest while methionine (0.31 g/100 g protein) was the lowest. The nonessential amino acids were also discovered, with glutamic acid (3.43 g/100 g protein) being the highest and glycine (0.38 g/100 g protein), the lowest. The study of the oxidizable vitamins revealed that vitamin C (1.33 mg/100 g) was more abundant than vitamin A (2.54 µg) and vitamin E (0.78 mg/100 g). This information will hopefully enhance the fruits acceptability by more people and thus, generally promote its utilization and appreciation in our diets.

## Introduction

*Synsepalum dulcificum* is a tropical fruit, native to West Africa. The plant belongs to the family – *Sapotaceae*. Although it can grow up to 20 feet high, its predominant form is shrubby. The plant first bears fruits after growing for approximately 2–3 years. At times, it produces two crops per year, often around March–April and later, after the rainy season. It produces green elongated leaves which remain green as long as they remain attached to the plant all year long (plate 1).

Although it has two varieties, distinguished by the production of red and yellow berries, the yellow variety (plate 1) is more prevalent in Nigeria, especially the Eastern part of Nigeria. The berry has a unique effect on the taste buds, such that flavors of fruits (citrus fruits), consumed after eating the fruit are generally enhanced and their delicate flavors, formerly masked by natural acids, are released, hence the name ‘miracle fruit’.

**Figure:**
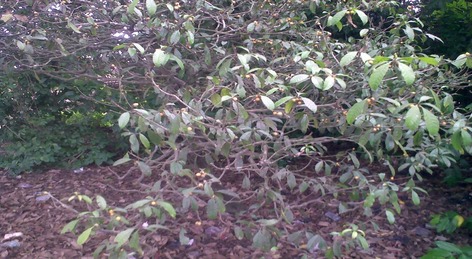


A new class of sweeteners from proteins found in the fruits of tropical plants has been discovered, and natives of the areas where the plants producing these proteins grow naturally have frequently used them to sweeten their food stuff. *Synsepalum dulcificum* is one of such plants. There is increased interest in natural sweeteners which may be as a result of ‘perceived’ health risks of some artificial sweeteners (WHO 1999). The miracle fruit has been in use since the 18th century (Slater 2007). Some scientists (Inglett et al. 1965) found some experimental evidence that the active principle was a macromolecule. The taste modifying principle was independently isolated by Kurihara and Beidler (1968), Henning et al. (1969), and Brouwer et al. (1968); and found to be miraculin. The destruction of the active principle by trypsin and pronase suggested its proteinaceous character. Other scientists (Metcalfe and Chalk 1972) from their studies confirmed that the sweetening property of miracle fruit was due to the presence of miraculin, a glycoprotein consisting of 191 amino acids and some carbohydrate chains (Theerasilp et al. 1989) found in the pulp of the berry. An evaluation of the amino acid profile of the yellow variety of *Synsepalum dulcificum*, therefore, becomes necessary to identify and quantify some of these amino acids. The vitamins content was investigated to determine if the pulp can provide additional benefits. This natural sweetener may be exploited especially by dieters and diabetes, who need more protein and vitamins in their diet.

## Materials and Methods

Fresh mature berries of *Synsepalum dulcificum* (miracle fruit) were obtained from Umuagwo in Ohaji Egbema Local Government Area of Imo State, Nigeria. The pulp of these freshly harvested and cleaned *Synsepalum dulcificum* berries (plate 2) was extracted by scraping the fruits with clean stainless spatula. It was oven dried and used for the vitamins and amino acid profile analysis.

### Determination of oxidizable vitamins content

The vitamins A, E, and C contents of the pulp sample were determined using the procedure described by Pearson (1976).

**Figure:**
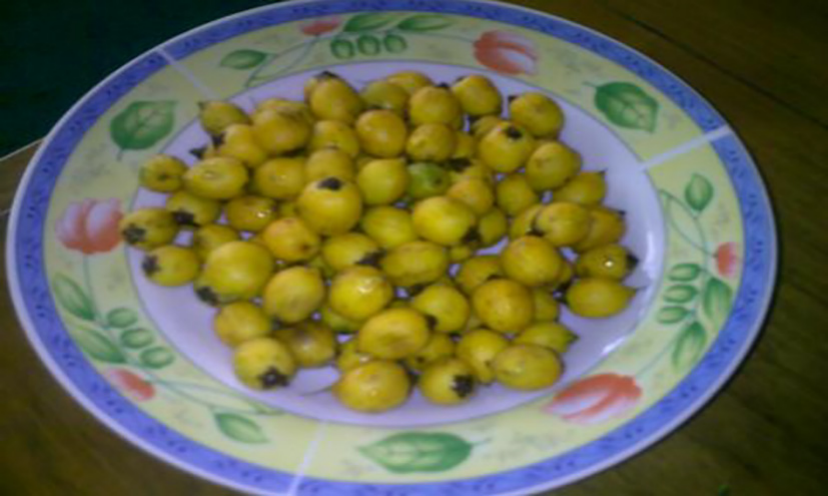


### Preparation of sample for vitamins determination

The sample was oven dried using a Baird and Tatlock oven BS2648 at a temperature of 50°C for 24 h.

### Determination of vitamin A content

**Figure 1:**
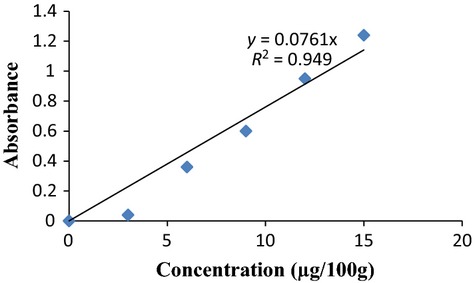
Standard curve of vitamin A concentrations.

One gram of the sample was macerated with 30 mL of absolute alcohol. Three milliliters of 50% potassium hydroxide was added. The solution was boiled for 30 min and cooled. Thirty milliliters of distilled water was added. The mixture was transferred to a separating funnel and washed with 10 mL of petroleum ether. The lower layer was discarded while the upper layer was evaporated to dryness. The residue was dissolved with 10 mL of isopropyl alcohol. The absorbance was taken at 334 nm using a spectrum 21D PEC spectrophotometer. The vitamin A content was extrapolated from a vitamin A standard curve (Fig.1). Alternatively, using the formula given below 

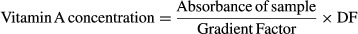
1

where DF = Dilution Factor

Gradient Factor = slope of the standard curve (Fig.1).

### Determination of vitamin E content

**Figure 2:**
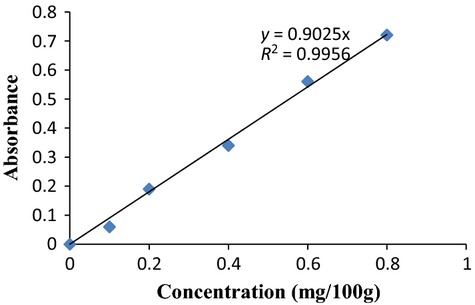
Standard curve of vitamin E concentrations.

One gram of the pulp sample was macerated with 20 mL of ethanol. The solution was filtered with Whatman No 1 filter paper. One milliliter of the filtrate was pipetted out and 1 mL of 0.2% ferric chloride in ethanol was added. One milliliter of 0.5% *α*-dipyridyl solution was also added. The solution was diluted to 5 mL with water and the absorbance read at 520 nm using a spectrum 21D PEC spectrophotometer. The vitamin E content was extrapolated from a vitamin E standard curve (Fig.2). Alternatively, using the formula given below: 

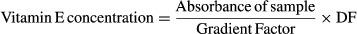
2

where DF = Dilution Factor

Gradient Factor = slope of the standard curve (Fig.2).

### Determination of vitamin C content

**Figure 3:**
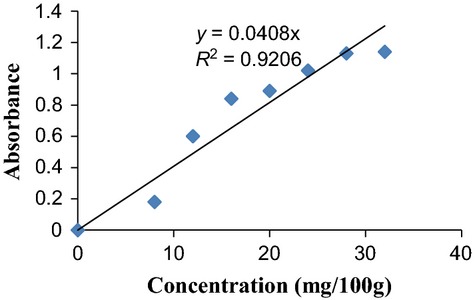
Standard curve of vitamin C concentrations.

One gram of the sample was macerated with 20 mL of 0.4% oxalic acid. It was filtered with Whatman No 1 filter paper. One milliliter of the filtrate was pipetted out and 9 mL of indophenol reagent added to it. The absorbance was read at 520 nm (using a spectrum 21D PEC spectrophotometer). The vitamin C content was extrapolated from a vitamin C standard curve (Fig.3). Alternatively, using the formula given below: 

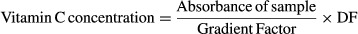
3

where DF = Dilution Factor

Gradient Factor = slope of the standard curve (Fig.3).

### Amino acid profile determination

The amino acid profile in the sample was determined using methods described by Speckman et al. (1958).

The sample was dried to constant weight. A known weight (300 mg) of the dried sample was put into extraction thimble and the fat was extracted using Soxhlet extraction apparatus as described by AOAC (2006). A small amount (200 mg) of ground fat-free sample was weighed, wrapped in Whatman No 1 filter paper, and put in a Kjeldhal digestion flask. It was digested and distilled. The distillate was then titrated with standardize 0.01 N hydrochloric acid to gray-colored end point and the percentage nitrogen in the sample was calculated using the formula below: 

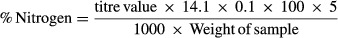
4

A known weight (50 g) of the defatted sample was put into glass ampoule. Seven milliliters (7 mL) of 6 N hydrochloric acid (HCl) was added and oxygen was expelled by passing nitrogen into the ampoule (this is to avoid possible oxidation of some amino acids such as methionine and cystine during hydrolysis). The glass ampoule was then sealed with Bunsen burner flame and put in an oven preset at 105°C ± 5°C for 22 h. The ampoule was allowed to cool before breaking it open at the tip and the content was filtered to remove the humins. The filtrate was then evaporated to dryness at 40°C under vacuum in a rotary evaporator. The residue was diluted with 5 mL acetate buffer (pH 2.0) and stored in plastic specimen bottles, which were kept in the freezer. It was noted that tryptophan was destroyed by hydrolysis with 6 N hydrochloric acid. Between 5 and 10 µL of the buffered residue was dispensed into the cartridge of the analyzer and analyzed with the TSM (Technicon Sequential Multi-sample) analyzer to acidic, neutral, and basic amino acids. The period of an analysis lasted for 76 min.

A constant ‘S’ was calculated for each amino acid in the standard mixture using the formula, 

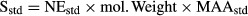
5where MAA = micromole of amino acid in the standard.

Finally, the amount of each amino acid present in the sample was calculated in g/16 gN or g/100 g protein using the following formula: 

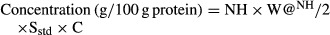
6

where: 

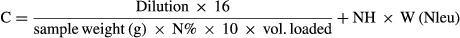
6.1

where: NH = Net height

W = Width @ half height

Nleu = Norleucine.

## Results and Discussion

From Table1, it was observed that although fruits are known as important sources of vitamins especially vitamins A and C, the pulp of yellow *Synsepalum dulcificum* was found to be very low in vitamin C, with content of 1.33 mg/100 g ± 0.24. This was less than the contents in other berries (blackberry, blueberry, raspberry, and strawberry) and fruits as reported by Food and Nutrition Board (2006), FNIC (2011) and Ihekoronye and Ngoddy (1985). Low ascorbic acid (vitamin C) levels have been associated with fatigue and increased severity of respiratory tract infections (Johnston et al. 1998). The vitamin A content of the sample was 2.54 µg (8.476 IU) (Table1). Although the precursors of vitamin A, including beta-carotene and certain other carotenoids are found particularly in yellow to orange colored fruits, the content in the sample does not compare favorably with the content in blackberry (214 IU), raspberry (160 IU), and blueberry (54 IU) (Wikipedia 2011a, 2011b, 2011c). The vitamin A value in the pulp was also very low compared to other fruits such as pineapple (50 IU), guava (200 IU), orange (120 IU), mango (1000–8000 IU), and pawpaw (2500 IU) (Harald 1997; Onyeka 2002; Food and Nutrition Board 2006). Deficiency of vitamin A leads to night blindness, failure of normal bone, and tooth development in the young and diseases of epithelial cells and membrane of the nose, throat, and eyes which decrease the body's resistance to infection (Arnold 1960). The vitamin E in the pulp of *Synsepalum dulcificum* (0.78 mg/100 g ± 0.05) (Table1) was higher than those of blueberry (0.57 mg) and raspberry (0.56 mg) (USDA 2004) but lower than the 1.17 mg content in blackberry (Wikipedia 2012). It was also higher than the content in the citrus fruits (0.24–0.25 mg) but lower than the content in mango (1.12 mg), pawpaw (1.12 mg), and avocado (1.34 mg) (Onyeka 2002). Vitamin E prevents the peroxidation of membrane phospholipids and cell membrane oxidation through its antioxidant actions. This berry is primarily consumed for its taste-modifying effect and not necessarily for its nutrients. As such, it is only eaten when there is a need for its sweetening function, making it highly underutilized. This investigation aims to change this by identifying its nutritional benefits. However, from the results above, it is observed that to adequately provide needed vitamins, in comparison with other berries and fruits, more quantity of the berry pulp may be consumed.

**Table 1:** Mean values of the vitamins identified in the pulp of *Synsepalum dulcificum* berry

Vitamin	Content
Vitamin A (*μ*g)	2.54 ± 0.27
Vitamin C (mg/100 g)	1.33 ± 0.24
Vitamin E (mg/100 g)	0.78 ± 0.05

Values are means of triplicate determinations.

All the essential amino acids were detected in the test sample (Table2). The chemical scores for the essential amino acids calculated from the WHO reference protein (Ihekoronye and Ngoddy 1985; Onuegbu et al. 2011) are also shown in Table2. The highest value was from leucine (2.35 g/100 g protein) with chemical score of 55.95%, followed by Lysine (1.60 g/100 g protein, chemical score of 38.10%), and the lowest from methionine (0.31 g/100 g protein) with chemical score of 14.09%. Leucine, isoleucine, and valine are oxidized in the muscle and the nitrogen used for the formation of alanine. All the analysed amino acids in miracle fruit had values lower than the amounts reported for African pear (*Dacryodes edulis*) pulp by FAO/WHO/UNU (1985). However, they were higher in quantity than the amino acids in *Pyrus communis* pear pulp (Mahammad et al. 2010). The nonessential amino acids were also detected as shown in table. Glutamic acid had the highest value (3.43 g/100 g protein) while glycine had the least value (0.38 g/100 g protein). Norleucine was not detected. The values of the amino acids – isoleucine, leucine, lysine, threonine, and valine – in the miracle berry, all exceeded the (FAO/WHO/UNU 1991) reference values of 2.8 mg/100 g protein, 6.6 mg/100 g protein, 5.8 mg/100 g protein, 3.4 mg/100 g protein, and 3.5 mg/100 g protein, respectively. The methionine + cysteine and phenylalanine + tyrosine (FAO/WHO/UNU 1991) reference values of 2.5 mg/100 g protein and 6.3 mg/100 g protein, respectively, were all exceeded in the miracle berry. This implied that the amino acids in the pulp of miracle fruit had high biological values and could contribute in meeting the human requirements of these essential amino acids especially if the commercial potential of this berry or its processed by-products is exploited. However, in comparison with the reference standard for ideal protein, the value for leucine and isoleucine contents of *Synsepalum dulcificum* pulp were below the recommended amino acid requirements (4.6 g/100 g protein) (Mahammad et al. 2010) for infants.

**Table 2:** Amino acid profile of *Synsepalum dulcificum* berry pulp

Amino acid	Amount g/100 g protein	Chemical score (%)
Lysine	1.60	38.10
Leucine	2.35	55.95
Isoleucine	0.82	19.52
Tyrosine	0.92	32.86
Phenylalanine	1.25	44.64
Threonine	0.52	18.57
Valine	0.52	12.38
Methionine	0.31	14.09
Proline	0.59	
Glycine	0.38	
Alanine	1.01	
Cystine	0.45	
Serine	0.77	
Glutamic acid	3.43	
Arginine	1.94	
Histidine	0.62	
Aspartic acid	1.76	

## Conclusion

The research revealed that the berry's pulp had more vitamin C than vitamins A and E. The oxidative vitamin content (vitamin C, A, and E) of the pulp was generally lower than that of other berries like blackberry, raspberry, and blueberry. The berry also had varying amounts of all the essential amino acids, with leucine having the highest amount and methionine the least value. This investigation on the yellow variety of the miracle berry has revealed the amino acid profile of the pulp. This study has also provided information on vitamin contents of the berry with respect to their identity and quantity in the pulp.

## Conflict of Interest

None declared.
